# Significantly different results in the ocular surface microbiome detected by tear paper and conjunctival swab

**DOI:** 10.1186/s12866-023-02775-3

**Published:** 2023-01-28

**Authors:** Zhangling Chen, Zhaoyu Xiang, Lipu Cui, Xinran Qin, Shuli Chen, Huiyi Jin, Haidong Zou

**Affiliations:** 1 Department of Ophthalmology, Songjiang Hospital Affiliated to Shanghai Jiao Tong University School of Medicine (Preparatory Stage), Shanghai, China; 2 Department of Ophthalmology, Shanghai General Hospital, Nanjing Medical University, Hongkou District, No. 100, Haining Road, Shanghai, 200080 China; 3 Department of Ophthalmology, Shanghai General Hospital, School of Medicine, Shanghai Jiao Tong University, Shanghai, China; 4 Shanghai Key Laboratory of Fundus Diseases, Shanghai, China; 5 Shanghai Eye Diseases Prevention & Treatment Center/Shanghai Eye Hospital, Shanghai, China; 6 National Clinical Research Center for Eye Diseases, Shanghai, China; 7 Shanghai Engineering Center for Precise Diagnosis and Treatment of Eye Diseases, Shanghai, China

**Keywords:** 16S rRNA, Tears, Conjunctiva, Microbes, Ocular surface

## Abstract

**Background:**

Great variation has been observed in the composition of the normal microbiota of the ocular surface, and therefore, in addition to differences in detection techniques, the method of collecting ocular surface specimens has a significant impact on the test results.The goal of this study is to ascertain whether the eye surface microbial communities detected by two different sampling methods are consistent and hence explore the feasibility of using tear test paper instead of conjunctival swabs to collect eye surface samples for microbial investigation.

**Materials and methods:**

From July 15, 2021, to July 30, 2021, nonirritating tear test strips and conjunctival swabs of both eyes were used in 158 elderly people (> 60 years old) (79 diabetic and 79 nondiabetic adults) in Xinjing Community for high-throughput sequencing of the V3-V4 region of the 16S rRNA gene. The composition of the microbial communities in tear test paper and conjunctival swab samples was analyzed.

**Results:**

There was no statistically significant difference in Alpha diversity of ocular surface microorganisms represented by tear strip and conjunctival swab in diabetic group (*P* > 0.05), but there was statistically significant difference in Alpha diversity of ocular surface microorganisms detected by tear strip and conjunctival swab in nondiabetic group (*P* < 0.05). There were statistically significant differences in Beta diversity of ocular surface microorganisms detected by two sampling methods between diabetic group and nondiabetic group (*P* < 0.05). There were statistically significant differences in ocular surface microorganisms detected by tear strip method between diabetic group and nondiabetic group (*P* < 0.05), but there was no statistically significant difference in conjunctival swab method (*P* > 0.05).

**Conclusions:**

Tear test paper and conjunctival swabs detect different compositions of microbes through two different techniques of eye surface microbe sampling. Tear test paper cannot completely replace conjunctival swab specimens for the study of microbes related to eye surface diseases.

## Introduction

The human conjunctival sac, as a semiopen cavity exposed to environmental factors in the long term, hosts a dynamic environment of microbiota throughout life, which typically do not cause abnormal or pathogenic conditions and are microecologically called the normal microbiota of the ocular surface, including the sum of many aerobic and anaerobic flora [1]. Small amounts of aerobic and anaerobic bacterial growth can be identified in naturally delivered neonates by ocular surface bacterial culture immediately after birth [2]. Under normal circumstances, air does not easily enter the conjunctival vault, readily causing a local relatively anoxic state, and the anoxic or hypoxic environment of Tenon’s capsule provides a favorable site for some anaerobic bacteria to parasitize the capsule. The overall abundance of microorganisms does not change with age, but the composition of the microbial community as a whole can change: the abundance of some phyla increases while that of others decreases [3]. The flora can also change in certain systemic disease states, such as the higher abundance of Acinetobacter on the ocular surface in diabetic patients compared to healthy, nondiabetic adults of the same age group [4].

Petrillo et al. showed [5] that the normal microbiota of the ocular surface was not pathogenic and that their presence may have an inhibitory effect on the overgrowth of pathogenic bacteria. However, an increasing number of studies have shown that once the healthy ocular surface microbiota was altered, they play an important role in the development of dry eye syndrome [6], trachoma [7], conjunctivitis [8], keratitis [9–11], etc. Miller [12] and Gupta [13] et al. sampled ocular surface microorganisms from patients with dry eye syndrome. After culturing was performed, they found an increased abundance of Staphylococcus aureus, coagulase-negative Staphylococcus and Corynebacterium, which were thought to be associated with the development of dry eye syndrome, possibly due to toxins produced by these bacteria [14, 15]. Our previous studies using 16S rRNA gene detection of ocular surface microbiota revealed the contribution of unclassified Clostridium spp. and Lactobacillus to the pathogenesis of diabetic dry eye [16], with the possible mechanism being the involvement of these bacteria in regulating the NF-kB and STAT-3 signaling pathways [17, 18] or in reducing lysozyme C and zinc-α-2-glycoprotein in the tear fluid [16].

Great variation has been observed in the composition of the normal microbiota of the ocular surface [19, 20], and therefore, in addition to differences in detection techniques, the method of collecting ocular surface specimens has a significant impact on the test results. Currently, tear test strips and conjunctival swabs are two commonly accepted methods of sample collection for studying ocular surface microorganisms, and some researchers [16, 21] use tear test strips to obtain test samples when performing ocular surface microbiology or genetic testing studies related to dry eye disease. In this process, one end of a sterile tear secretion test strip is placed in the conjunctival sac of the subject and the other end is suspended from the lid margin and left for 5 min before removing the test strip with sterile scissors. This method is also used in tear proteomics studies [22]. Other researchers have selected conjunctival swabs for microbial culture in some microbiological studies related to ocular infectious diseases, where a sterile swab is used to wipe the conjunctiva of the upper and lower fornix of the subject under surface anesthesia while avoiding contact with the cornea and lower eyelid margin, keeping the head end of the swab for microbial culture or genetic testing analysis [7, 8, 23]. Therefore, is a tear paper specimen, or a conjunctival swab specimen, more representative of the distribution of microorganisms associated with the ocular surface? To date, no uniform standard is available for ocular surface microbial collection, probably because there have been no previous studies comparing the results of both sampling methods on the same individuals.

Characterization of ocular surface microbiota can be used to accurately analyze the role of microbiota in the development of ocular diseases and to select targeted drugs for the purpose of treating ocular diseases to precisely adjust the microecological balance and re-establish an environment conducive to ocular surface health [24]. To this end, we randomly selected 79 healthy, nondiabetic individuals and 79 diabetic patients from elderly residents without ocular surface disease in the community, with the aim of understanding the differences in the ocular surface microbiota test results obtained from two different collection methods within the same eye. We then explored the reasons for the differences and provided a basis for future studies on accurate analysis of the ocular surface microbiota.

## Materials and methods

From July 15, 2021, to July 30, 2021, older adults with confirmed diabetes and nondiabetic older adults from the Shanghai Cohort Study of Diabetic Eye Disease (SCODE), an annual epidemiological survey of eye disease in the Xinjing Community of Shanghai, were included in our study. The inclusion criteria for the study subjects were as follows: (1) subjects were fully informed about the study and signed a written informed consent form; (2) subjects were all > 60 years old; (3) subjects were able to cooperate in completing the eye examination and specimen collection; and (4) the diagnostic criteria for diabetic patients were met in accordance with the World Health Organization (WHO) diagnostic criteria [25]. Exclusion criteria for study subjects included the following: (1) presence of eyelid disorders: impingement, entropion, incomplete closure of eyelid defect, etc.; (2) presence of dry eye, conjunctival disease and corneal disease; (3) presence of other ocular diseases, such as cataracts and retinopathy; (4) complications from serious chemical damage to the eye, history of previous serious trauma to the eye; (5) history of eye surgery within 3 months and history of corneal contact lens wear; (6) current treatment with eye drops; and (7) presence of hypertension, heart disease, systemic lupus erythematosus, dry syndrome, Grave's disease, etc.

All subjects enrolled in this screening were arranged in a room with suitable light, temperature and humidity for tear test paper (Jingming, Tianjin, China; 40 × 5 mm) and conjunctival swab (GeWei Bio-Tech (Shanghai) Co.Ltd) specimen collection after completing all eye examinations. The sampling method was as follows: the patient's eyelid skin was wiped twice with a cotton swab soaked in saline before sampling. The tear sampling method was first selected by collecting tears from both eyes of each subject separately with tear paper. The moistened portion of the tear paper from both eyes was removed with sterile scissors and placed as one specimen in a test tube containing DNA protective solution. The specimen was then quickly placed in a -20 °C refrigerator for freezing and storage. After the tear specimen collection was completed, the patient was allowed to rest as needed, and then the conjunctival swab sampling method was used, in which the upper and lower conjunctival sacs of both eyes were wiped with different sides of the swab, and the head of the swab was left as a swab specimen. The specimens were quickly placed in a tube containing DNA protective solution and stored in a -20 °C refrigerator for freezing. DNA extraction was completed immediately after all specimens were collected.

All subjects were scheduled to have their eye examinations and specimens collected at Xinjing Community Health Center. Ocular specimens were collected by the same trained ophthalmologist, Z.C., to ensure the uniformity of the results.

A power and paired sample size estimator based on the permutational multivariate analysis of variance (PERMANOVA) application Micropower [26], assessed that, similar to the previous ocular microbiome study [27] for low abundance 16S rRNA datasets, a minimum sample size of 30 was required to produce a discriminant power of 0.8 with a significance level of 0.05. Therefore, we aimed for a minimum of 40 subjects per study group, and 79 subjects were taken from each of the DM (diabetes mellitus) and non-DM groups in this study to meet the required sample size for the study. Statistical processing was next performed using SPSS 22.0 software. Age, BMI and fasting glucose were compared between the two groups of adults using the independent samples t test; the chi-square test was used for sex comparisons.

### Microbiological testing and data analysis

The collected tear test strips and conjunctival swab specimens were extracted according to the kit instructions. Total microbial genomic DNA was extracted from all specimens using an OMEGA Soil DNA Kit (M5635-02) (Omega Bio-Tek, Norcross, GA, USA), and the total microbial genomic DNA was stored in a refrigerator at -20 °C for further analysis. The quality and quantity of DNA were determined by agarose gel electrophoresis and a NanoDrop NC-2000 spectrophotometer, respectively. The V3-V4 region of the bacterial 16S rRNA gene was amplified by PCR using forward primer 338F (5'-ACTCCTACGGGAGGCAGCA-3') and reverse primer 806R (5'-GGACTACHVGGGTWTCTAAT-3'), and the sample-specific tag sequences (7 bp (base pairs)) were incorporated into the primers for multiplex sequencing.

After the above steps were completed, equal amounts of amplification products were mixed together, and double-end 2 × 250 bp sequencing was performed using the Illumina MiSeq platform and MiSeq Reagent Kit v3 program. Sequence data analysis was performed mainly using QIIME2 2019.4 and the R package (v3.2.0). The Greengenes database was used to compare the ASV (amplicon sequence variant) signature sequences with the reference sequences in the database to obtain the taxonomic information corresponding to each ASV. The ASVs with abundance values lower than 0.001% of the total sequencing of all samples were removed, the total number of sequences in each sample in the ASV abundance matrix was randomly sampled at different depths, and the sparse curve was drawn with the number of sequences drawn at each depth and their corresponding ASVs. The ASV abundance matrix was randomly flattened to the lowest 95% of the amounts of sequences in all samples, thus correcting for the sequencing depth-induced diversity differences between samples. The following seven diversity indices were calculated separately for each group of samples using QIIME2(2019.4) software, including the Chao1 index, Faith's PD index, Good' s coverage index, the Shannon index, the Simpson index, Pielou's evenness index and the observed species index, and box line plots were drawn to compare the richness and evenness of ASV between different groups of samples. Beta diversity was analyzed using the UniFrac distance metric to investigate changes in microbial community structure between samples. The compositional profiles of species based on the genus level were examined by principal coordinate analysis (PCoA) and nonmetric multidimensional scaling analysis (NMDS). PERMANOVA was used to evaluate the significance of differences in microbial community structure between groups. The linear discriminant analysis effect size (LDA effect size, LEfSe) method was used to detect taxonomic units rich in differences between groups. This is a method that combines nonparametric Kruskal‒Wallis and Wilcoxon rank sum tests with linear discriminant analysis effect size. LDA effect size allows direct analysis of differences at all categorical levels simultaneously, with differences considered statistically significant at *P* < 0.05.

## Results

From July 15, 2021 to July 30, 2021, a total of 160 older adults, including 80 diabetic and 80 healthy, nondiabetic individuals, met the inclusion criteria for this study in the annual epidemiological survey of ophthalmology in the Xinjing Community of Shanghai in the SCODE study. All 160 subjects completed specimen collection by tear test paper, and 158 of these subjects only completed specimen collection by the conjunctival swab method. Therefore, 158 subjects were finally recruited in this study, including 79 diabetic patients and 79 healthy, nondiabetic individuals, and the two groups were assigned into the DM (including DM-T tear group and DM-S swab group) group and non-DM (including NDM-T tear group and NDM-S swab group) group. The number of both groups met the sample size requirement, and the basic profiles of the two groups were as follows: the mean ages of the DM group and the non-DM group were 67.47 ± 6.26 years and 68.68 ± 4.56 years, respectively, with no significant difference (*P *> 0.05). There were 39 and 34 males and 40 and 45 females in the two groups, respectively, with no statistical significance (*P* > 0.05). The BMIs of the two groups were 24.55 ± 3.17 and 24.64 ± 3.39, respectively, with no significant difference (*P* > 0.05). Fasting blood glucose was 6.99 ± 2.18 mmol/L in the DM group and 5.53 ± 0.79 mmol/L in the non-DM group, and the difference was statistically significant (t = 5.60, *P* < 0.05). The DM and non-DM groups were matched with each other in terms of sex, age and BMI; the differences were not statistically significant (*P* > 0.05), and the differences in fasting glucose were statistically significant (*P* < 0.05).

The distribution of ASV characteristic sequences/OTU representative sequences in the DM and non-DM groups was mainly between 404–431 bp in length, with an average of 425 bp per sequence, and 99.9% of the high-quality sequences had lengths distributed between 400 and 431 bp. Primer removal, mass filtration and chimera removal were carried out by the DADA2 method, and there was no statistical significance in the total number of effective sequences obtained between the DM group and the non-DM group (t = 1.43, *P* > 0.05). In the DM group, the total number of effective sequences was 9,129,441 (reads), and the total number of high-quality sequences was 7,569,884 (reads), accounting for 82.9%. The total number of effective sequences in the DM-S group was 10,388,711 (reads), and the total number of high-quality sequences was 8,341,766 (reads), accounting for 80.3%. In the non-DM group, the total number of effective sequences in the NDM-T group was 9,509,444 (reads), and the total number of high-quality sequences was 7,994,088 (reads), accounting for 84.1%. The total number of effective sequences in the NDM-S group was 9,621,626 (reads), and the total number of high-quality sequences was 7,680,882 (reads), accounting for 79.8%. The proportion of high-quality sequence quantities of tear paper was slightly higher than that of conjunctival swabs in both groups, and the difference was statistically significant (t = 2.13 and t = 4.46, *P* < 0.05).

**Fig. 1:**
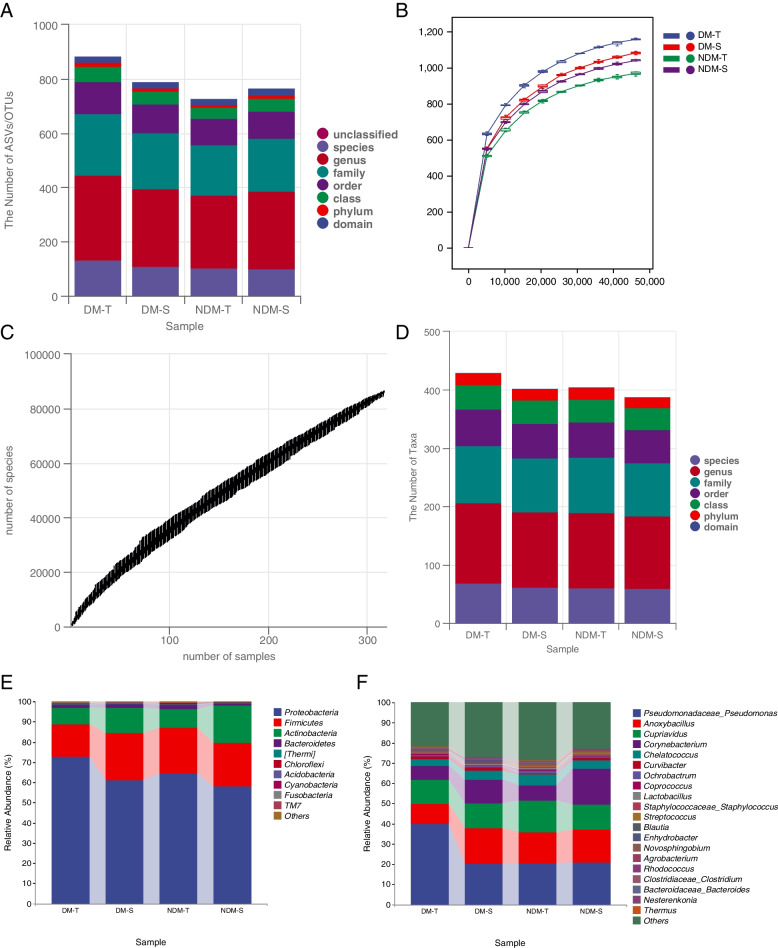
**A** The number of ASVs/OTUs in the four study groups; **B** Rarefaction Curve; **C** Species accumulation curves; **D** The number of taxa in four groups; **E** The relative abundance of four groups at phyla level (top 10); **F** The relative abundance of four groups at genus level (Top 20)

The high-quality sequences were grouped into ASVs/OTUs (operational taxonomic units) according to 98% sequence similarity, and the total ASVs/OTUs were calculated for each group at each taxonomic level of the domain, phylum, class, order, family, genus and species. A total of 884 ASVs/OTUs were annotated to the DM-T group, 790 to the DM-S group, 728 to the NDM-T group, and 768 to the NDM-S group (Fig. 1A). The results showed that there was a difference in the number of ASVs/OTUs annotated by the two sampling methods in the two different groups. In the DM group, the ASVs/OTUs annotated in the tear group were higher than those in the swab group, and in the NDM group, the ASVs/OTUs annotated in the swab group were higher than those in the tear group. A total of 1674 ASVs/OTUs were annotated in the DM group, which was significantly higher than the 1496 ASVs/OTUs in the NDM group.

The flatness of the sparsity curve (Fig. 1B) reflected the magnitude of the effect of sequencing depth on the diversity of microbial communities. In this study, the sparsity curve gradually leveled off as the amount of sequencing data increased, indicating that the current sequencing depth was sufficient to reflect the richness and evenness of the microorganisms contained in this sample. The species accumulation curve (Fig. 1C) showed that the sample size was sufficient for this study.

The specific composition of microbial communities in each sample at each taxonomic level could be obtained by counting the ASVs/OTUs after resolving the differences induced by different depths of sampling. It was possible to calculate the number of taxonomic units contained in different groups at each taxonomic level (Fig. 1D), from which we can see that at the phylum, class, order, family, genus and species levels, both the DM and non-DM groups had more species detected in the tear group, with the DM group having a somewhat higher number of species detected than in the non-DM group.

Comparing the ocular bacterial taxon composition of the subjects in each group, the bacterial 16S rRNA sequences of the individual subjects were classified into the phylum level and genus level. At the phylum level (Fig. 1E), Proteobacteria abundance in the DM group was significantly higher than that in the non-DM group, while Actinobacteria abundance was significantly lower than that in the non-DM group. Proteobacteria, [Thermi], Chlorobacteria and Acidobacteria detected in tear test paper in the DM group and non-DM group were higher than those in the conjunctival swab tests of both groups, while Actinobacteria detected in tear test paper was significantly lower than that in the conjunctival swabs (QIIME2(2019.4) software).

At the genus level, most of the 16S rRNA gene sequencing results of ocular surface bacteria in each group were classified into 20 genera (Fig. 1F): Pseudomonas, Anoxybacillus, Cupriavidus, Corynebacterium, Chelatococcus, Curvibacter, Ochrobactrum, Coprococcus, Lactobacillus, Staphylococcus, Streptococcus, Blautia, Enhydrobacter, Novosphingobium, Agrobacterium, Rhodococcus, Clostridium, Bacteroides, Nesterenkonia, Thermus, etc. Corynebacterium in the DM group was lower than that in the non-DM group, while Curvibacter in the DM group was higher than that in the non-DM group. In both the DM group and non-DM group, the numbers of Ochrobactrum and Coprococcus in tear paper were higher than those in the conjunctival swabs, while the numbers of Anoxybacillus, Corynebacterium and Curvibacter were higher in the conjunctival swabs than those in the tear paper. The number of Pseudomonas detected on tear paper in the DM group was significantly higher than that in the conjunctival swab group (QIIME2(2019.4) software).

**Fig. 2:**
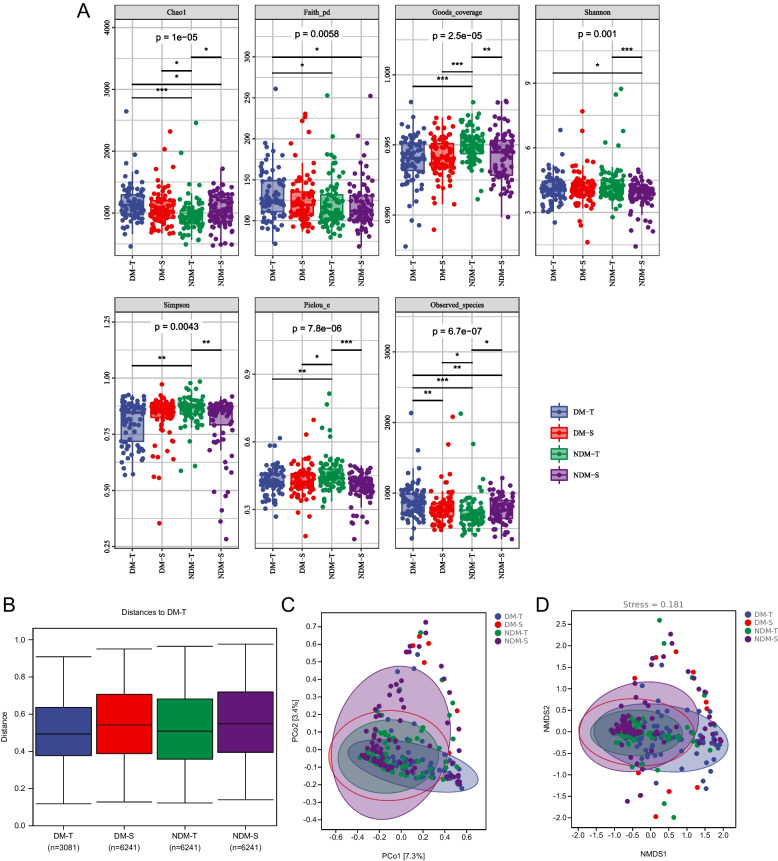
**A** Alpha diversity index analysis of the four study groups; **B** Analysis of differences between the four groups; **C** PCoA analysis; **D** NMDS analysis

Our results showed that the alpha diversity indices of ocular surface microorganisms detected by tear test paper and conjunctival swabs in the DM group were not significantly different except for the observed species index (*P* > 0.05), revealing no differences in the abundance, uniformity and coverage of ocular surface microorganisms extracted by either tear test paper or conjunctival swabs in the DM group. In contrast, the alpha diversity indices of ocular surface microorganisms detected by tear paper and conjunctival swabs in the non-DM group were significantly different except for the Faith-pd index (*P* < 0.05), suggesting differences in the abundance, uniformity and coverage of ocular surface microorganisms extracted by tear paper and conjunctival swabs in the non-DM group. The alpha diversity indices of ocular surface microorganisms detected by tear paper in both groups were significantly different except for the Shannon index (*P* < 0.05), indicating that the microorganisms extracted by tear paper differed in abundance, uniformity, and coverage, whereas the microorganisms extracted by conjunctival swabs in both groups did not differ in abundance, uniformity, and coverage. (Fig. 2A, Table 1).

**Table 1:** Alpha diversity index analysis

Alpha	Group							
	Alpha diversity index				*p*-value			
					DM-T vs DM-S	NDM-T vs NDM-S	DM-T vs NDM-T	DM-S vs NDM-S
	DM-T	DM-S	NDM-T	NDM-S				
Chao1	1158.407	1082.59	969.121	1040.365	0.088	0.04*	0.000003*	0.65
Faith_pd	131.338	125.713	120.222	120.018	0.39	0.86	0.011*	0.43
Goods_coverage	0.994	0.994	0.995	0.994	1	0.0014*	0.0001*	1
Shannon	4.126	4.109	4.36	3.825	0.65	0.00047*	0.39	0.11
Simpson	0.805	0.83	0.859	0.8	0.49	0.0093*	0.01*	0.49
Pielou_e	0.423	0.428	0.459	0.4	0.57	0.000002*	0.0047*	0.06
Observed_species	882.82	790.819	728.38	768.59	0.0055*	0.035*	0*	0.84

^*^p < *0.05(Kruskal–Wallis rank sum test)*

The PLS-DA discriminant model based on the relative abundance at the species level was constructed by the unweighted UniFrac distance algorithm, with NMDS dimension taken as 2 and elliptical confidence level 0.95. The.

PCoA analysis and NMDS analysis are shown in Fig. 2C and D. In this study, the ocular surface microbes represented by two different sampling methods across all participants showed differences. Meanwhile, the ocular surface microbes detected by the same sampling method in the DM group and the non-DM group also showed differences.

Using the Bray–Curtis distance matrix file, "PERMANOVA" analysis for intergroup differences was performed with the scikit-bio package in Python, and the differences in microorganisms detected by both tear paper and conjunctival swabs in the DM and non-DM groups were statistically significant (Fig. 2B and Table 2). Differences were found between the DM and non-DM groups for the tear paper method but not for the conjunctival swab method. Again, this indicates that there were differences in the ocular surface microbiota represented by the two different sampling methods.

**Table 2:** Analyses of differences between the four groups

Group1	Group2	Samplesize	Permutations	pseudoF	*p*value	qvalue
all	-	316	999	8.146913	0.001*	-
DM-T	DM-S	158	999	13.25696	0.001*	0.002
NDM-T	NDM-S	158	999	3.773281	0.01*	0.015
DM-T	NDM-T	158	999	14.011523	0.001*	0.002
DM-S	NDM-S	158	999	1.828771	0.127	0.127

^*^p < *0.05(PERMANOVA analysis)*

**Fig. 3:**
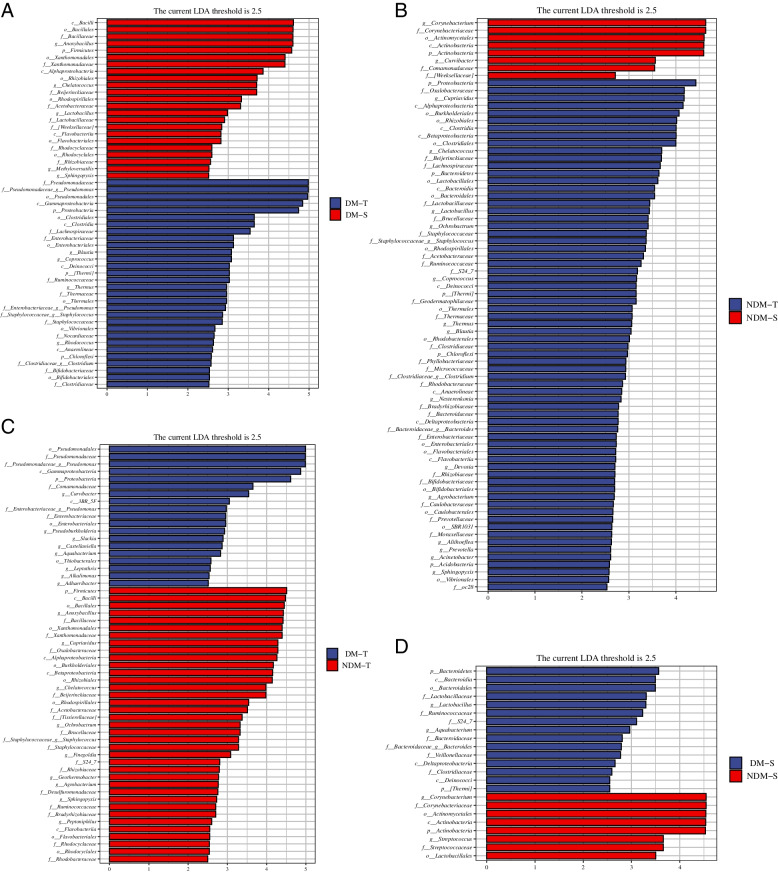
Species with significant differences between the two groups(*P *< 0.05): **A** LEfSe analysis in DM-T and DM-S group; **B** LEfSe analysis in NDM-T and NDM-S group; **C** LEfSe analysis in DM group and NDM group with tear test paper; **D** LEfSe analysis in DM group and NDM group with conjunctival swabs

LEfSe analysis was used to further compare the species composition differences between the tear paper and conjunctival swab methods in the DM group and the non-DM group, and marker species with significant differences were obtained (Fig. 3A, B, C, and D). In the non-DM group, tears were more abundant with Proteobacteria, Bacteroidetes, [Thermi], Chloroflexi, Acidobacteria and Cyanobacteria at the phylum level (*P* < 0.05), while only Actinobacteria abundance in swabs was significantly different (*P* < 0.05). Tear test paper showed that the levels of Cupriavidus, Chelatococcus, Lactobacillus, Ochrobactrum, Staphylococcus, Coprococcus, Thermus, Blautia, Clostridium, and Bacteroides and the high abundance of Acinetobacter were significantly different (*P* < 0.05), and conjunctival swabs showed a significant difference in the high abundance of Corynebacterium and Curvibacter (*P* < 0.05). Compared with the non-DM group, the abundance of Proteobacteria, [Thermi] and Chloroflexi was higher in the DM group, as was the abundance of Firmicutes (*P* < 0.05). Tear test paper showed significant differences in the levels of Pseudomonas, Blautia, Coprococcus, Thermus, Staphylococcus, and Rhodococcus and a high abundance of Clostridium (*P* < 0.05). Conjunctival swabs showed significant differences in Corynebacterium and Chelatococcus and a high abundance of Lactobacillus (*P* < 0.05).

The abundance of Proteobacteria detected in the tear test was significantly higher in the DM group than in the non-DM group (*P* < 0.05), and the abundance of Firmicutes in the non-DM group was significantly higher than that in the DM group (*P* < 0.05). The abundance of Pseudomonas and Curvibacter was significantly higher in the DM group than in the non-DM group (*P* < 0.05), while the abundance of Anoxybacillus, Cupriavidus, Chelatococcus, Ochrobactrum, Staphylococcus, and Agrobacterium was significantly higher in the DM group than that in the non-DM group (*P* < 0.05). The abundance of Bacteroidetes and [Thermi] detected by conjunctivitis swabs in the DM group was significantly higher than that in the non-DM group (*P* < 0.05), while the abundance of Actinobacteria in the non-DM group was significantly higher than that in the DM group (*P* < 0.05). At the genus level, the abundance of Lactobacillus, Aquabacterium and Bacteroidetes in conjunctival swabs in the DM group was significantly higher than that in the non-DM group (*P* < 0.05), while the abundance of Corynebacterium and Streptococcus in the non-DM group was significantly higher than that in the DM group (*P* < 0.05).

## Discussion

The normal microbiota of the ocular surface refers to a range of nonpathogenic microorganisms that reside on the conjunctiva and cornea, including the core microbiota of the ocular surface and the microbiota only temporarily present [28]. Growing numbers of studies have shown [23, 28–31] that microbiota play an important role in ocular health and disease, that alterations in the ocular surface microbiota are risk factors for many ocular diseases and that comprehensive evidence of the ocular surface microbiota is important for both the prevention and treatment of numerous ocular diseases. However, there is no uniform standard for the collection of ocular surface microorganisms. A search of previous articles studying ocular surface microbiota revealed only two sampling methods, with more studies using conjunctival swab sampling to explore ocular surface microbiota but fewer applying tear test strips. The different collection methods resulted in findings that were not completely uniform [12, 22, 23, 32, 33]. For example, Dong et al. [28] found a high abundance of Actinobacteria on the eye surface of 4 healthy Caucasian people through a conjunctival swab study. Huang et al. [19] found a high abundance of Actinobacteria on the eye surface of 31 healthy people in Shandong Province, China, through a conjunctival swab study. Zhang et al. [16] found a relatively high abundance of Firmicutes on the surface of 22 healthy human eyes in Shanghai, China, through a tear test paper study. To date, no studies have been designed to compare the results of different ocular surface sampling methods for ocular microbiological testing in the same cohort.

This study is the first to evaluate the consistency of the results obtained from two sampling methods by testing the microorganisms of specimens collected in a community population. In our epidemiological study, the community is more broadly representative of the elderly population, who are more likely to have ocular and other systemic diseases, including diabetes mellitus. Compared to children and young adults, the elderly are more likely to cooperate with us for conjunctival swabs because of their relatively insensitive ocular surface. Therefore, the elderly population in the community was chosen as the study population for this study. Considering that, in addition to the age factor, diabetes is a common disease affecting the structure of ocular surface flora abundance [4, 16], two groups of elderly people, diabetic and nondiabetic, were finally included in the present study, and tear test strips and conjunctival swabs from the ocular surface were completed. All specimens were tested with 16S rRNA gene sequencing. This method is more mature in the field of eye surface microbe research and is more economical than metagenomics, which is suitable for the study of large populations in epidemiology.

The low level of ocular surface microorganisms makes it difficult to collect specimens [34], but with a standardized sampling process and 16S rRNA gene sequencing, it is possible to obtain clear results for flora analysis. The present study showed that more high-quality sequences were detected by tear test strips in the same cohort, and more species were annotated. The composition of the main microorganisms detected at the phylum level by tear test strips and conjunctival swabs in the nondiabetic population as well as in the diabetic population is consistent with that in previous studies [5, 16, 27, 28] and mainly includes Proteobacteria, Firmicutes and Actinobacteria. This finding indicates that at the phylum level, both tear test strips and conjunctival swabs reflect the normal core microbial composition of the ocular surface. However, there were still significant differences in the relative abundance of major microbial organisms at the phylum level between the test results obtained by these two methods, such as Actinomycetes and Proteobacteria. Some differences were also observed in the composition and relative abundance of species at the genus level. Alpha diversity and beta diversity analyses of the microorganisms obtained by the two sampling methods in the two groups showed that there were differences in microbial diversity between the two sampling methods, indicating that the choice of sampling method is very important when studying microbial species with very low ocular surface content. (Table 3).

**Table 3:** Comparison of the two different sampling methods used for microbial investigation

Sampling methods	Selected sampling region	Influence Factors	Advantages	Disadvantages	Appropriate sampled subjects
Tear paper	Lower conjunctival sac, eyelid margin and lower eyelid skin	Lysozyme, lactoferrin, and defensins	Non-invasive, easy to perform	Time consuming, susceptible to tear volume and environment	All age
Conjunctival swab	The upper and lower conjunctival sac	Mucin, goblet cells, and conjunctival epithelial cells	Less time used for sampling	Risk of trauma	Adults

Possible reasons for the difference in the detection results of the two sampling methods include the following: 1. Regarding the sampling site, tear fluid contains microorganisms from the conjunctival sac, the eyelid margin and possibly from the skin, and previous studies have shown [1, 20, 31, 35] that there are significant differences in microorganisms from the ocular surface, eyelid margin, and skin. The presence of environmental microorganisms cannot be ignored due to the exposure of tear test strips to air. The conjunctival surface is covered with tear film, and the conjunctival swabs contain a certain amount of tear microorganisms [16]. Conjunctival swabs avoid contact with the cornea and lower eyelid margin and are less time-consuming for collecting specimens. They also reduce the influence of environmental microorganisms. Thus, it appears that conjunctival swabs are a more representative sampling method of the ocular surface microbiota. However, swab sampling can vary depending on the strength of the operator, and previous studies have shown [28, 36, 37] that the depth of swabbing can also affect the composition of the microorganisms collected. 3. The role of the specific components of the tear and conjunctival swabs should not be overlooked; tear fluid contains lysozyme, lactoferrin and defensins that have a destructive effect on microorganisms [34], and while the conjunctival sac as a semienclosed cavity provides a more ideal site for microbial growth and colonization, conjunctival sac secretions also contain a certain amount of antimicrobial components [38], such as mucin and conjunctival cupped cells. These factors caused the differences found in the results of this study at the phylum and genus level. For example, at the phylum level, the abundances of Firmicutes and Bacteroides in the non-DM group were significantly higher in tear test paper than in conjunctival swabs, which may be related to the fact that Staphylococcus and Lactobacillus are more common on the skin of the eyelids and stained during tear test paper sampling [39]. As another example, at the genus level, anaerobic Bacillus and Corynebacterium were significantly lower in tear test strips than in conjunctival swabs, which may be because these bacteria are affected by the hypoxic environment in the conjunctival sac.

DM is a metabolic disorder dominated by hyperglycemia, which has a significant impact on ocular surface diseases such as dry eye and corneal neuropathy. In our previous study, we used tear test strips only for diabetic dry eye [16], and the results of the tear test strips in the present study were generally consistent with those of our previous study. The detection of ocular surface flora on conjunctival swabs in the DM population was also reported for the first time in this study, and the results were found to be very different between the two sampling methods. We believe that this was related to the hyperglycemic microenvironment and abnormal glucose metabolism in DM patients. For example, we found that Pseudomonas were more likely to multiply in tears with high sugar content, and Anoxybacillus were instead prone to die. For another example, due to peripheral neuropathy caused by high glucose, diabetic patients are prone to eyelid lesions, leading to the easy growth of Staphylococcus on the eyelid surface, which is reflected in an increased abundance in tear test paper. At present, the specific mechanism of differences in ocular surface flora caused by diabetes is still not completely clear and needs further study. We hypothesize that a series of changes related to abnormal glucose metabolism could cause ocular surface abnormalities and affect the abundance of microflora. For example, advanced glycation end-products (AGEs) are produced in large quantities in high glucose concentrations. AGEs can be deposited not only in the lacrimal gland and conjunctiva but also in the basement membrane or matrix of the corneal epithelium. The accumulation of AGEs destroys the structure of the lacrimal gland, damages the goblet cells of the conjunctiva, and leads to reduced tear secretion and decreased tear film stability [40–42]. These changes in the ocular surface microenvironment may cause the difference in test results of conjunctival swabs and tear strip samples.

In addition to considering the factors that lead to differences in the flora obtained between the two collection methods due to the different sampling sites, researchers in practice must consider the acceptance of the different sampling methods by the sampled subjects. Traditional conjunctival sampling of ocular lesions is often performed by swabbing mainly for simplicity, speed, and ease of use, and this method is less painful and more acceptable to patients when operated on under surface anesthesia, but it is uncertain whether this method is suitable for microbiological sampling in all ocular disease studies. When there is no obvious lesion in the eye or when the collection of ocular surface microorganisms needs to be performed on a healthy population, the single wipe site and the need to operate under surface anesthesia may affect the results of the detection of true ocular surface microorganisms [28, 43, 44]. For some studies investigating ocular surface microorganisms in children, such sampling methods often make it difficult for children to comply or are not acceptable to parents. Therefore, some researchers have considered using tear specimens instead of swab specimens for the study of ocular surface microorganisms. It is believed that tear samples are not only easy to collect, preserve and transport but also contain high-quality DNA [16, 45–48].

Tears are distributed on the surface of the eye and represent the characteristics of the entire ocular surface. The link between the pathogenesis of dry eye and the quality and quantity of tears has been clear, and apart from inflammatory factors, the influence of microorganisms on tears should not be ignored. Zhang [16] et al. conducted a study of ocular surface microorganisms in dry eyes and concluded that it is more reasonable to use tear test paper than conjunctival swabs. In the case of combined ocular infections, which often produce large amounts of secretions in the conjunctival sac, conjunctival swabs allow targeted sampling and can be repeated, making it easier to achieve specimen volume. Ocular infectious diseases often require faster test results, and the use of swab sampling can satisfy culture conditions, whereas tear fluid is often not used due to low levels of bacteria.

This study has some shortcomings. Only elderly Han Chinese people were enrolled in this study; only non-diabetic and diabetic people were compared, and only 16S rRNA comparisons were made. Regardless of the sampling method, the findings could be affected by the volume of the collected specimens because the amount of tear secretion will influence the test results, especially in elderly patients with dry eyes and extremely low tear secretion. In addition, the depth, site and scope of the swab test wipe and the use of surface anesthetics may also affect the microbial content of the collected specimens.

## Conclusion

Our finding that different sampling methods can be used to detect different compositions of eye surface microbes in the same individual provides a new perspective for the study of the eye surface microbiome. At the same time, this study contributes to the consideration of adopting a more standardized sampling method, and it is hoped that in the future, researchers can fully consider the particularity of ocular structure and choose a better sampling method. Tear collection may pose comparatively smaller damage, and it is also commonly used in the diagnosis of diseases, which is easily accepted by patients, especially children, but more time is needed for the tear test; therefore, it is not suitable for taking samples in a large population. Although conjunctival sac swabs are convenient, the damage they may cause is larger, making them merely suitable for adults and for a study of ocular surface microbes with a large sample size. Overall, we recommend that in future studies on ocular surface microbes, it would be advisable to use a combination of tear strips and conjunctival swabs for a more accurate location of ocular surface microbes.

## Data Availability

The detail data and materials in the current study are available from the corresponding author Prof. Haidong Zou(zouhaidong@sjtu.edu.cn) on reasonable request or use the following links ( http://www.ncbi.nlm.nih.gov/bioproject/913284).
